# The Ulcerative Effects of Levamisole-Induced Vasculitis

**DOI:** 10.7759/cureus.90012

**Published:** 2025-08-13

**Authors:** Angela Zavaro, Azary Hernandez, Nadin Abboud, Seth Bleicher

**Affiliations:** 1 Internal Medicine, Jackson Memorial Hospital, Miami, USA; 2 Internal Medicine, American University of the Caribbean School of Medicine, Miami, USA

**Keywords:** c-anca vasculitis, cocaine adulterated with levamisole, cocaine levamisole-induced vasculitis, drug-induced cutaneous vasculitis, malnutrition, polysubstance use disorder

## Abstract

Levamisole-induced vasculitis is an autoimmune vasculitis that is most commonly associated with the use of levamisole-adulterated cocaine. We present a case of a 35-year-old undomiciled woman who was admitted to the inpatient psychiatric unit for drug detoxification and evaluation of bilateral lower extremity ulcerations. Her clinical presentation, along with a history of cocaine use, raised suspicion for levamisole-associated vasculitis. Symptomatic resolution and cutaneous healing in these cases are typically observed within 2-14 months following sustained abstinence from cocaine. This case highlights the growing concern for the lack of routine screening of drug adulterants such as levamisole in patients with known polysubstance abuse. To improve outcomes and reduce morbidity and mortality in this patient population, routine laboratory evaluation and diagnostic workup for adulterating agents should be incorporated into standard protocols. Additionally, comprehensive treatment plans should include nutritional support, wound care, and individualized addiction management strategies. Early recognition and targeted intervention are essential for optimizing care and promoting recovery in patients with substance use-related complications.

## Introduction

Cocaine use in the United States has devastatingly transpired into a nationwide crisis. It is estimated that over two million people suffer from cocaine addiction. Cocaine's chemical properties interfere with the limbic system in the brain, anatomically located in the medial portion of the forebrain. This interconnected network is responsible for producing motivation and pleasure [1]. Cocaine further inhibits dopamine reuptake, leading to surplus dopamine neurotransmitters in the nucleus accumbens, amplifying euphoric sensation and pleasure. As cocaine use continues, the brain's reward system is reinforced with an intensely strong association with the drug. Street cocaine is commonly mixed with other substances, such as levamisole.

Levamisole is an anthelmintic medication that was discontinued in 1999 for use in humans due to its adverse effects of leukopenia, agranulocytosis, and skin vasculitis [2]. However, levamisole is still found in about 70% of today's cocaine distribution [2]. It enhances the stimulatory effect of cocaine by inhibiting monoamine oxidase and catechol-O-methyltransferase activity, prolonging the duration of catecholamines in the synapse, and increasing the reuptake inhibition effect of cocaine [2]. Cocaine induces vasoconstriction through several mechanisms, including the blockade of norepinephrine reuptake. Norepinephrine plays a crucial role in the sympathetic nervous system, as it acts on alpha-1 adrenergic receptors, leading to vasoconstrictive properties in the smooth muscle cells of blood vessels. Additionally, cocaine further releases a potent vasoconstrictor known as endothelin-1 from endothelial cells, as well as increasing the influx of calcium across endothelial cell membranes.

Moreover, cocaine's chemical ability to induce vasoconstriction manifests in substantial deficits in vital bodily functions. The most prominent insufficiency of the catastrophic effects of cocaine abuse is the decrease in blood supply, transpiring as a substantial deficiency of oxygen and nutrients to organs [3].

The patient in this case report is a regular cocaine user who has been exposed to levamisole, further exacerbating c-ANCA (cytoplasmic antineutrophil cytoplasmic antibodies) vasculitis. This is an autoimmune condition in which the immune system generates antibodies that attack healthy blood vessels, ultimately leading to inflammation and occlusion of healthy blood flow [4]. Vasculitis can destroy blood vessels in organs such as the lungs and kidneys, in turn damaging them [5]. The antibodies created by the body's immune system are directed against an enzyme recognized as proteinase-3, located in neutrophils [2].

Additionally, this patient has multiple comorbidities that have transpired to poor health management. Significantly, the patient had low serum albumin, further delaying curative measures. Reduced serum albumin levels may result from severe malnutrition or hepatic dysfunction, which can be a result of chronic liver diseases such as hepatitis. Albumin plays a critical role in the wound healing process, as it is imperative in the transportation of nutrients, maintaining fluid balance, and contributing to tissue repair [6].

## Case presentation

**Figure 1:**
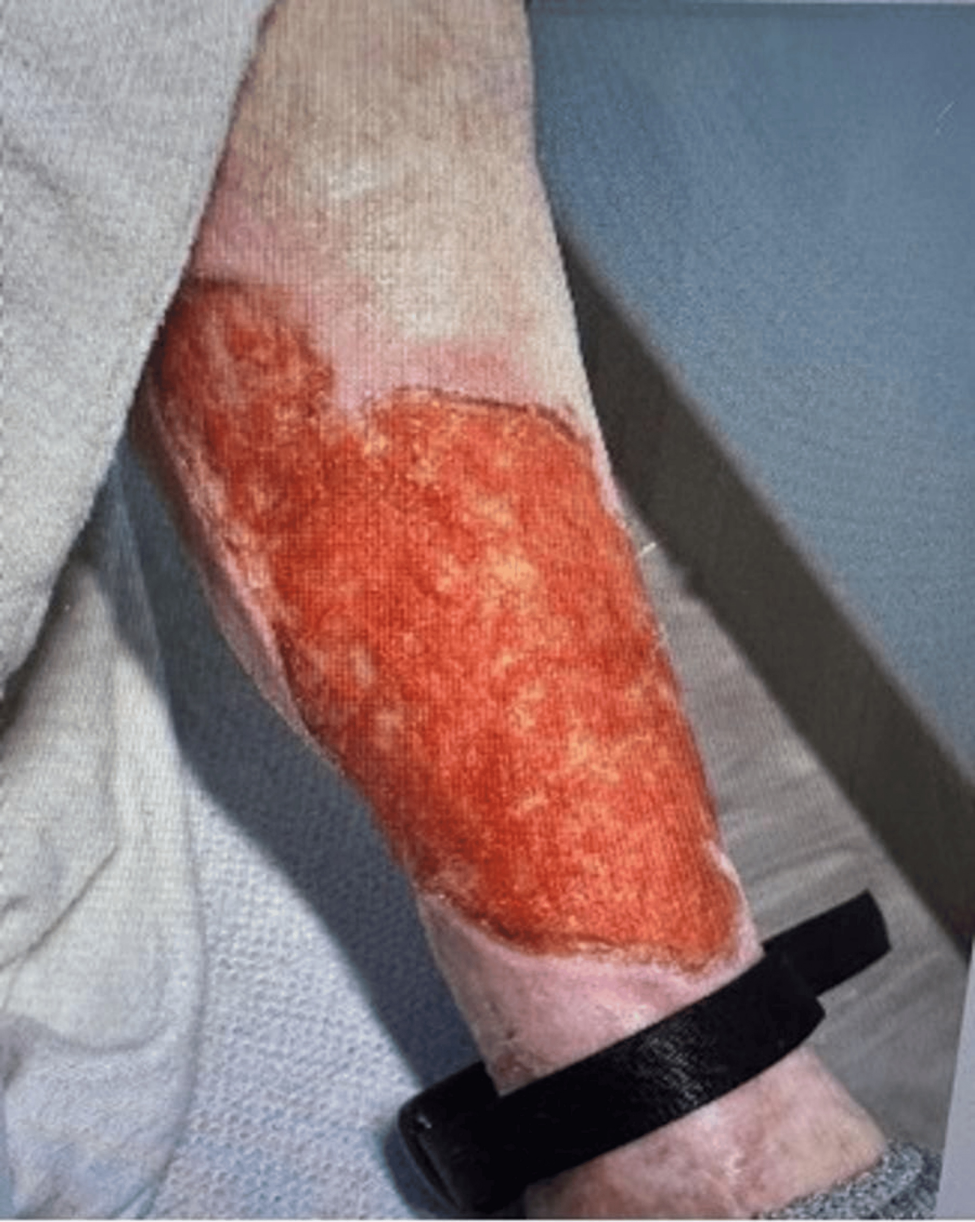
Lower extremity ulcerative lesion.

**Figure 2:**
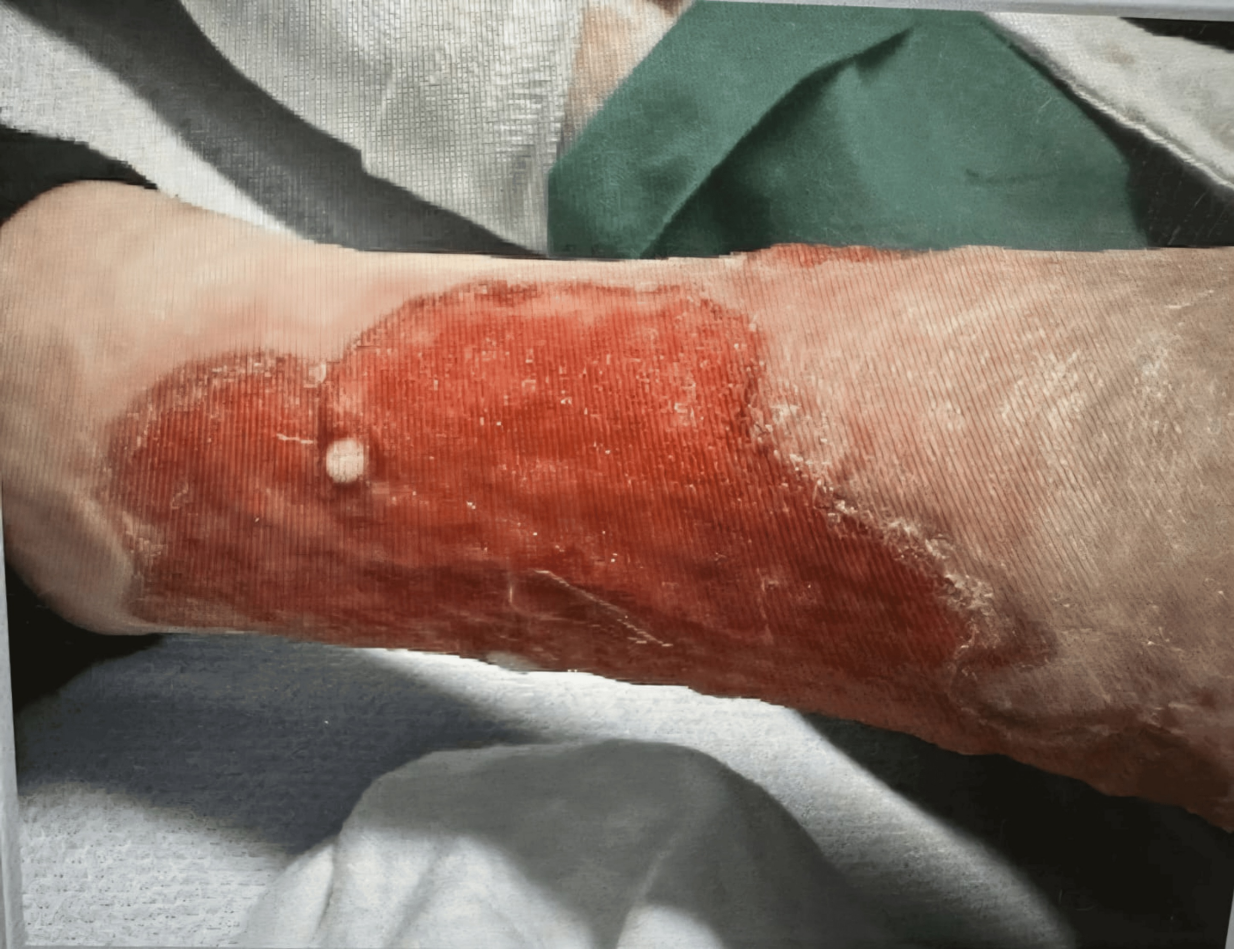
Leg ulcer with necrotic core and hypopigmented lesion.

The patient is an undomiciled 35-year-old female presenting with chronic bilateral leg ulcerations and is admitted into an inpatient psychiatric unit for drug detoxification. She has a past medical history of HIV, hepatitis C virus, deep vein thrombosis, syphilis, c-ANCA-positive vasculitis, and polysubstance abuse, specifically cocaine and fentanyl. A physical examination revealed a systolic murmur on a cardiovascular assessment and poor dentition with multiple missing teeth. Examination of the extremities demonstrated wound dressings saturated with purulent yellow drainage. Dermatologic inspection revealed ulcerative lesions on the lower extremities with necrotic centers and hypopigmented lesions (Figures 1, 2). The patient denied any pain, burning, itching, fevers, chills, or night sweats.

Laboratory results are summarized in Table 1, with the patient's values compared against standard reference ranges. The decreased serum albumin may reflect underlying hepatic dysfunction, possibly related to liver disease, and/or severe malnutrition. Although the patient is HIV positive, an undetectable viral load indicates effective viral suppression with antiretroviral therapy.

**Table 1:** Patient's laboratory values on admission c-ANCA: cytoplasmic antineutrophil cytoplasmic antibodies

Parameters	Patient Values	Reference Range
Albumin	2.7 g/dL	3.5–5.5 g/dL
CD4+	353 cells/mm³	500–1500 cells/mm³
Viral load	Undetectable	Undetectable: <50 copies/mL
c-ANCA	Positive	Negative or less than 2.8 units/mL
Cocaine	Positive	Negative

**Figure 3:**
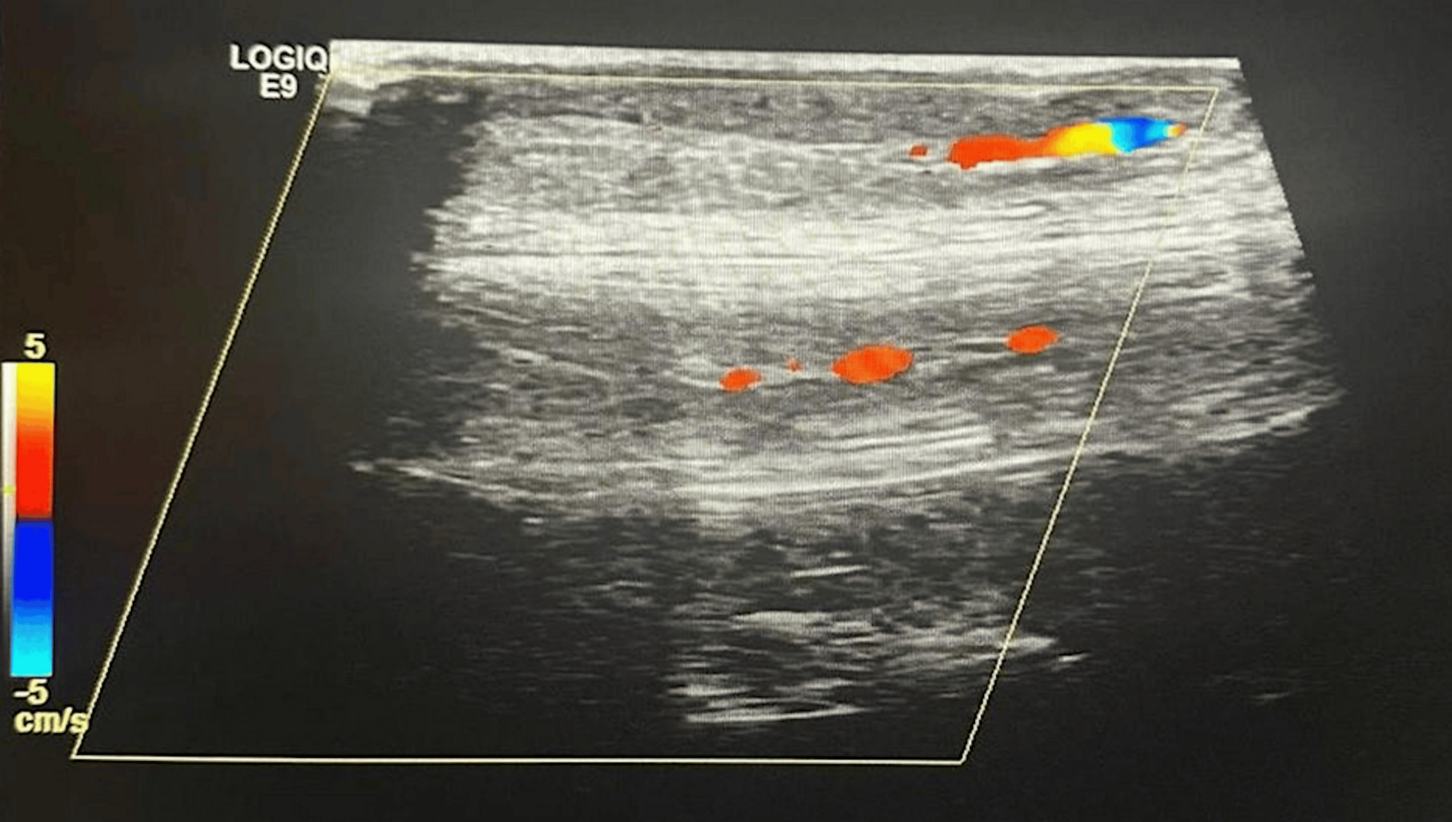
Occlusion of the right posterior tibial artery with minimal blood flow leading to decreased oxygen delivery, ulcer formation, and impaired wound healing of the right lower leg.

The presence of both cocaine and c-ANCA supports the diagnosis of drug-induced small-vessel vasculitis. Lower extremity arterial ultrasound findings show occlusion of the right anterior tibial artery. Furthermore, occlusion of the right posterior tibial artery with minimal blood flow is visualized in Figure 3. Multiple collateral vessels in the left distal anterior and posterior tibial arteries due to chronic occlusion have formed.

Treatment includes maintenance of her HIV medications, drug detoxification, and customized wound care, including Xeroform gauze (petrolatum and 3% bismuth tribromophenate) and clean wrapping. With sustained cessation of cocaine use, clinical improvement and healing of ulcerative lesions are typically observed within 2-14 months, indicating a favorable prognosis. This patient was discharged with her medication regimen and clean gauze. Since discharge on this admission, the patient has demonstrated poor compliance with follow-up appointments.

## Discussion

This case highlights the complex interaction between levamisole-induced vasculitis, polysubstance abuse, specifically cocaine, and comorbidities such as HIV, hepatitis C virus, c-ANCA-positive vasculitis, and malnutrition. Street cocaine compounded with levamisole constricts the vasculature and inhibits perfusion, resulting in ulcerations and necrosis. This presents a diagnostic challenge due to overlapping symptoms of vasculitis, drug toxicity, and malnutrition. Managing this patient requires addressing not only the vasculitis but also the substance abuse and nutritional deficiencies, including low albumin, that impair wound healing [6].

Although this condition is rare, urgent attention is required. Cocaine is known to be the second most popular illegal recreational drug in the United States, behind marijuana. Moreover, other toxic substances are commonly added to cocaine. Many narcotic medications include other potentially toxic agents that are known to cause worsening health and are not routinely tested in the clinical setting. This leads to increased morbidity and mortality, especially in patients with known disease(s). Access to drug screening panels that include the possibility of adulterants, in combination with public health departments, can revise screening measures to maximize patient care through the evolving drug crisis.

Adequate albumin levels in the body contribute to fluid balance, as well as antioxidant and anti-inflammatory protection of cells. Moreover, albumin plays a substantial role in the process of wound healing, as it is composed of amino acids. Amino acids are vital for collagen synthesis, fibroblast proliferation, and tissue proliferation. Hypoalbuminemia is linked to the prolongation of the inflammatory phase, decreasing fibroblasts, suppressing collagen synthesis and can be a poor prognostic marker of health status. Inadequate levels of albumin in this patient's laboratory workup have exemplified complications and delays of the healing process, mainly due to her chronic hepatitis diagnosis and lack of maintenance of her healthcare regimen as an undomiciled patient.

Chronic hepatitis damages the liver and impairs albumin production, as well as HIV. Although this patient's HIV load is undetectable, the chronic hepatitis, along with polysubstance abuse and malnutrition (lack of protein consumption), is the suspected main determinant factor explaining the protracted complications of her leg ulcerations. Management of hypoalbuminemia should indicate correcting any underlying cause of inflammation prior to supplementation of albumin via infusion [7].

## Conclusions

The prognosis of this 35-year-old female patient is favorable with adherence to her current HIV regimen, cocaine cessation, nutritional improvement, and supportive care. The chronic bilateral leg ulcerations are anticipated to have a partial restoration of blood flow with the cessation of both levamisole and cocaine. Since occlusion is present, further intervention may also be necessary, such as stent placement, to potentially restore blood flow in her distal lower extremities.

There is adequate research material regarding levamisole-induced vasculitis; however, additional research is indicated for how malnutrition can further worsen prognosis, especially in the setting of substance abuse of one or more offending agents. Additionally, more research is necessary regarding the worsening of health outcomes due to toxic agents that are not routinely tested. Since many patients admitted to medical facilities have substance abuse issues, including cocaine, further laboratory/diagnostic testing, customized assessments, and treatment plans are necessary to provide optimal care. Rehabilitation facilities for polysubstance abuse and close monitoring post-discharge from inpatient hospital settings can additionally optimize patient compliance through case management. Clinically, this case highlights the importance of a thorough social history, past medical history, early intervention, and a multidisciplinary approach to treating the underlying vasculitis, promoting wound healing, and ultimately improving patient outcomes.
